# Molecular origins of mutational spectra produced by the environmental carcinogen *N*-nitrosodimethylamine and S_N_1 chemotherapeutic agents

**DOI:** 10.1093/narcan/zcad015

**Published:** 2023-03-27

**Authors:** Amanda L Armijo, Pennapa Thongararm, Bogdan I Fedeles, Judy Yau, Jennifer E Kay, Joshua J Corrigan, Marisa Chancharoen, Supawadee Chawanthayatham, Leona D Samson, Sebastian E Carrasco, Bevin P Engelward, James G Fox, Robert G Croy, John M Essigmann

**Affiliations:** Department of Chemistry, Massachusetts Institute of Technology, Cambridge, MA 02139, USA; Department of Biological Engineering, Massachusetts Institute of Technology, Cambridge, MA 02139, USA; Center for Environmental Health Sciences, Massachusetts Institute of Technology, Cambridge, MA 02139, USA; Division of Comparative Medicine, Massachusetts Institute of Technology, Cambridge, MA 02139, USA; Department of Chemistry, Massachusetts Institute of Technology, Cambridge, MA 02139, USA; Department of Biological Engineering, Massachusetts Institute of Technology, Cambridge, MA 02139, USA; Center for Environmental Health Sciences, Massachusetts Institute of Technology, Cambridge, MA 02139, USA; Department of Chemistry, Massachusetts Institute of Technology, Cambridge, MA 02139, USA; Department of Biological Engineering, Massachusetts Institute of Technology, Cambridge, MA 02139, USA; Center for Environmental Health Sciences, Massachusetts Institute of Technology, Cambridge, MA 02139, USA; Department of Chemistry, Massachusetts Institute of Technology, Cambridge, MA 02139, USA; Department of Biological Engineering, Massachusetts Institute of Technology, Cambridge, MA 02139, USA; Center for Environmental Health Sciences, Massachusetts Institute of Technology, Cambridge, MA 02139, USA; Department of Biological Engineering, Massachusetts Institute of Technology, Cambridge, MA 02139, USA; Center for Environmental Health Sciences, Massachusetts Institute of Technology, Cambridge, MA 02139, USA; Department of Biological Engineering, Massachusetts Institute of Technology, Cambridge, MA 02139, USA; Center for Environmental Health Sciences, Massachusetts Institute of Technology, Cambridge, MA 02139, USA; Department of Chemistry, Massachusetts Institute of Technology, Cambridge, MA 02139, USA; Department of Biological Engineering, Massachusetts Institute of Technology, Cambridge, MA 02139, USA; Center for Environmental Health Sciences, Massachusetts Institute of Technology, Cambridge, MA 02139, USA; Department of Chemistry, Massachusetts Institute of Technology, Cambridge, MA 02139, USA; Department of Biological Engineering, Massachusetts Institute of Technology, Cambridge, MA 02139, USA; Center for Environmental Health Sciences, Massachusetts Institute of Technology, Cambridge, MA 02139, USA; Department of Biological Engineering, Massachusetts Institute of Technology, Cambridge, MA 02139, USA; Center for Environmental Health Sciences, Massachusetts Institute of Technology, Cambridge, MA 02139, USA; Department of Biology, Massachusetts Institute of Technology, Cambridge, MA 02139, USA; Division of Comparative Medicine, Massachusetts Institute of Technology, Cambridge, MA 02139, USA; Laboratory of Comparative Pathology, Memorial Sloan Kettering Cancer Center, Weill Cornell Medicine, and The Rockefeller University, New York, NY 10065, USA; Department of Biological Engineering, Massachusetts Institute of Technology, Cambridge, MA 02139, USA; Center for Environmental Health Sciences, Massachusetts Institute of Technology, Cambridge, MA 02139, USA; Department of Biological Engineering, Massachusetts Institute of Technology, Cambridge, MA 02139, USA; Center for Environmental Health Sciences, Massachusetts Institute of Technology, Cambridge, MA 02139, USA; Division of Comparative Medicine, Massachusetts Institute of Technology, Cambridge, MA 02139, USA; Department of Chemistry, Massachusetts Institute of Technology, Cambridge, MA 02139, USA; Department of Biological Engineering, Massachusetts Institute of Technology, Cambridge, MA 02139, USA; Center for Environmental Health Sciences, Massachusetts Institute of Technology, Cambridge, MA 02139, USA; Department of Chemistry, Massachusetts Institute of Technology, Cambridge, MA 02139, USA; Department of Biological Engineering, Massachusetts Institute of Technology, Cambridge, MA 02139, USA; Center for Environmental Health Sciences, Massachusetts Institute of Technology, Cambridge, MA 02139, USA

## Abstract

DNA-methylating environmental carcinogens such as *N*-nitrosodimethylamine (NDMA) and certain alkylators used in chemotherapy form *O*^6^-methylguanine (m6G) as a functionally critical intermediate. NDMA is a multi-organ carcinogen found in contaminated water, polluted air, preserved foods, tobacco products, and many pharmaceuticals. Only ten weeks after exposure to NDMA, neonatally-treated mice experienced elevated mutation frequencies in liver, lung and kidney of ∼35-fold, 4-fold and 2-fold, respectively. High-resolution mutational spectra (HRMS) of liver and lung revealed distinctive patterns dominated by GC→AT mutations in 5’-Pu-G-3’ contexts, very similar to human COSMIC mutational signature SBS11. Commonly associated with alkylation damage, SBS11 appears in cancers treated with the DNA alkylator temozolomide (TMZ). When cells derived from the mice were treated with TMZ, *N*-methyl-*N*-nitrosourea, and streptozotocin (two other therapeutic methylating agents), all displayed NDMA-like HRMS, indicating mechanistically convergent mutational processes. The role of m6G in shaping the mutational spectrum of NDMA was probed by removing MGMT, the main cellular defense against m6G. MGMT-deficient mice displayed a strikingly enhanced mutant frequency, but identical HRMS, indicating that the mutational properties of these alkylators is likely owed to sequence-specific DNA binding. In sum, the HRMS of m6G-forming agents constitute an early-onset biomarker of exposure to DNA methylating carcinogens and drugs.

## Graphical Abstract

**Graphical Abstract:**
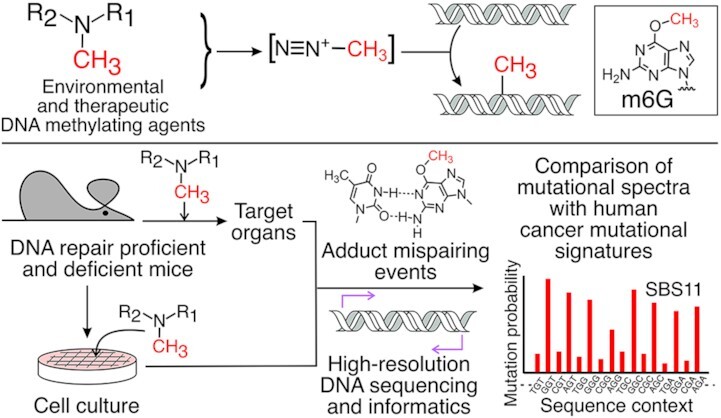
Distinct DNA methylating agents, including *N*-nitrosodimethylamine and temozolomide generate nearly identical mutational spectra in repair proficient and deficient mice, and in cell culture, suggesting a convergent chemical mechanism and mutational process.

## INTRODUCTION

**Figure 1.**
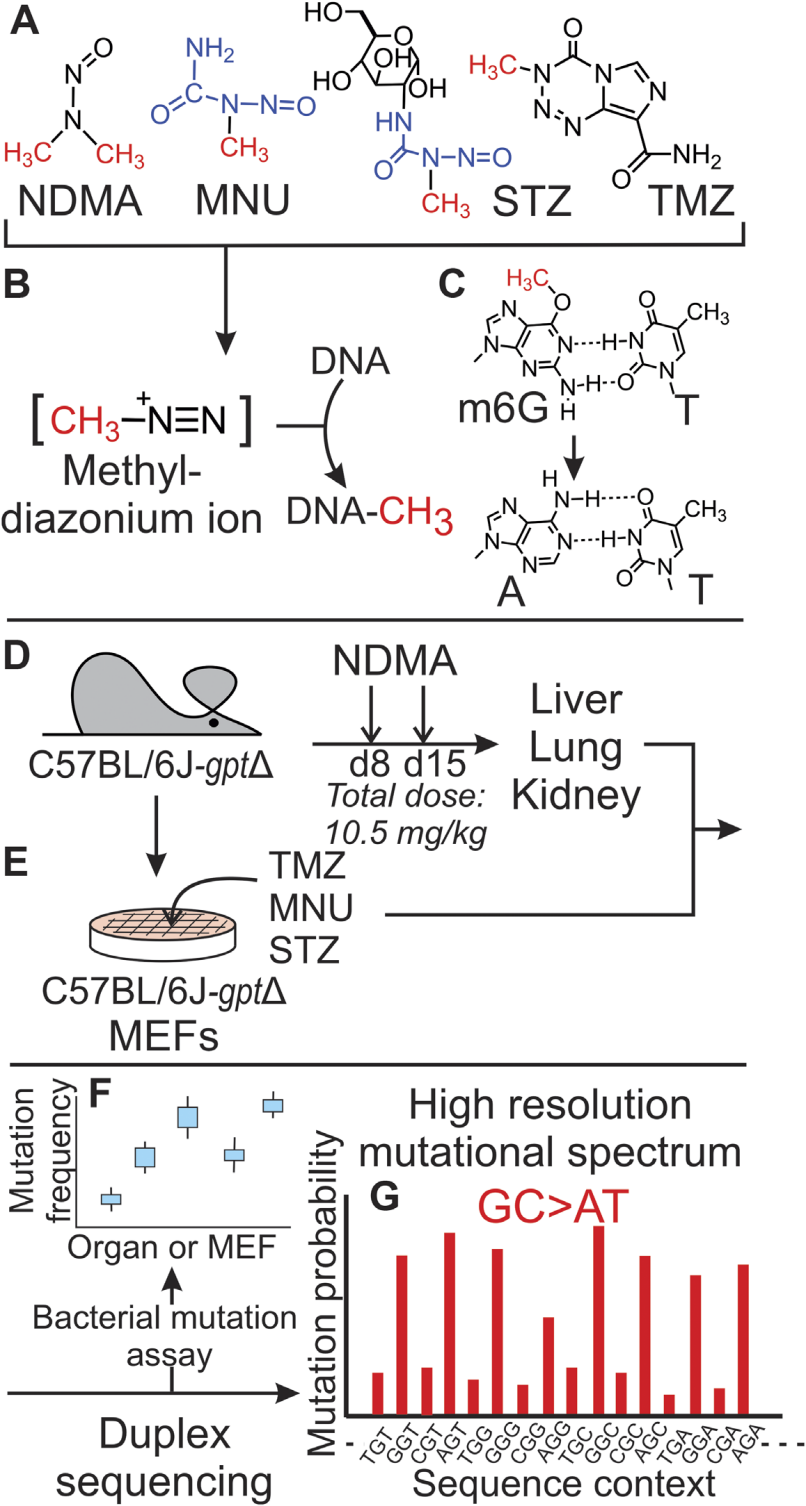
Experimental workflow from toxicant exposure to high-resolution mutational spectra (HRMS). (**A**) Compounds evaluated for mutagenic properties include *N*-nitrosodimethylamine (NDMA), *N*-methyl-*N*-nitrosourea (MNU), streptozotocin (STZ) and temozolomide (TMZ), all of which form a putative methyldiazonium ion (**B**) prior to reaction with DNA to form adducts such as *O*^6^-methylguanine (m6G) (**C**), a mutagenic adduct that mispairs with thymine during DNA synthesis. Portions of MNU and STZ are differentially colored in blue to show shared structural features, and the red functional group on each structure denotes the methyl group transferred to DNA. (**D**) Neonatal C57BL/6J-*gpt*Δ mice were treated with NDMA on days 8 and 15 post-birth for a total dose of 10.5 mg/kg. Lung, liver and kidney were harvested 10 weeks following the second injection. In some experiments, derivatives of the C57BL/6J-*gpt*Δ mouse were used, where the *RaDR* locus was incorporated with or without the *Mgmt* gene, which encodes a protein that repairs m6G. (**E**) In parallel, mouse embryo fibroblasts (MEFs) generated from C57BL/6J- *gpt*Δ mice were treated with the direct-acting mutagens, MNU, STZ or TMZ, as indicated. DNA isolated from these samples was analyzed: (**F**) for point mutations in the *gpt* gene, or (**G**) subjected to duplex sequencing to generate an HRMS for each agent.

*N*-Nitrosamines such as *N*-nitrosodimethylamine (NDMA; Figure 1) are DNA alkylating agents that are abundant in the human environment (1,2). The toxic, mutagenic and carcinogenic effects of NDMA have been demonstrated in numerous animal studies (3,4), leading to classification of this agent by the International Agency for Research on Cancer as a probable (Group 2A) human carcinogen (5). While human exposure can occur through several routes, industrial contamination of watersheds has been a major source of concern, particularly for NDMA (6). This *N*-nitrosamine has been detected as a contaminant of drinking water at locations on the Environmental Protection Agency National Priorities List, including a Superfund Site in Wilmington, Massachusetts, where the agent was recently associated with a cluster of childhood malignancies (7,8). Risk of exposure, however, is not limited to industrial sites because *N*-nitrosamines are also found in cured meats (9,10), tobacco combustion products (11), and contaminated pharmaceuticals, including valsartan, metformin, and ranitidine (12–15).

Like many carcinogens, NDMA requires biochemical activation to a DNA damaging electrophile as a chemical prelude to its potent biological effects. As such, DNA alkylation by NDMA and other *N*-nitroso compounds is believed to be mediated by reactive alkyl diazonium ions. The electrophilic methyldiazonium ion (Figure 1B) derived from NDMA is the biotransformation product of the parent carcinogen by mammalian cytochrome P450s, primarily isoenzyme 2E1 (CYP2E1) (16,17). Reaction of the methyldiazonium ion with DNA produces 7-methylguanine (m7G; ∼70% of all DNA adducts), methyl phosphotriester (a backbone adduct; ∼15%), *O*^6^-methylguanine (m6G; ∼7%), 3-methyladenine (m3A; ∼3%), and several other quantitatively minor DNA base adducts (18–20).

DNA damaging electrophiles can be classified by the degree of selectivity they display for oxygens during nucleophilic substitution reactions, with S_N_1 agents showing more oxyphilicity than agents that react via an S_N_2 mechanism (21). When one considers methylation reactions, S_N_1 agents react not only with the most nucleophilic targets (e.g. the N7 of guanine), but also with base and phosphate oxygens, including the *O*^6^- of guanine and the *O*^4^- and *O*^2^- of thymine. Reactions at the oxygen atoms on nucleobases, especially the *O*^6^- of guanine, are commonly associated with potent mutagenicity and carcinogenicity (22–26). Among the many methylated DNA bases, m6G has a special significance in that it plays two distinct and important roles in the cellular response to DNA alkylating agents. First, its ability to pair with thymine during DNA synthesis (Figure 1C) (27) leads to an m6G:T base pair, which is a target for mismatch repair enzymes that convert the T-containing strand into a nick that eventually matures into a lethal strand break (28,29). This property explains the mechanism underlying several cytotoxic agents that are used or have been used in cancer chemotherapy, including the *N*-methyl nitrosamides *N*-methyl-*N*-nitrosourea (MNU) and streptozotocin (STZ), and the widely used methyl imidazotetrazine, temozolomide (TMZ) (30–32). Second, if a cell escapes the toxic effect of the m6G:T pairing event, an additional round of replication generates GC→AT mutations (29,33–36), which are often seen in the wake of exposure to cancer-causing DNA alkylating agents, as shown in the current work with the environmental carcinogen, NDMA. The distinctive roles m6G plays in environmental carcinogenesis, as well as in cancer therapy, underscore the importance of finding ways to classify DNA alkylating agents by the patterns of DNA adduct-driven mutations they form in living cells.

Several biochemical systems dictate sensitivity or resistance to agents such as NDMA. As one example, DNA repair systems offer defenses against several DNA adducts and the loss or exceeded capacity of these defenses enhances cancer risk (37–39). Many studies with repair-deficient cells or animals show that the levels of specific methylated DNA adducts typically track with mutagenic and carcinogenic endpoints, pointing to the role that these adducts could have in malignant transformation and the role that specific repair systems have to prevent cancer initiation (40,41). With agents such as NDMA, a second factor that determines risk is the level of expression of enzymatic systems that generate the DNA-interactive metabolites. For example, in liver tissue, which is a prime target for neoplastic transformation by NDMA, hepatocytes in the centrilobular regions are notably vulnerable to DNA adduct formation, in part because they are among the first cells encountered following most methods of exposure to alkylating agents and in part because they typically have high CYP2E1 expression (42,43). Lung and kidney also express this CYP, albeit to a lower extent, and are also target tissues (44,45).

The current work compared mutations induced by NDMA, MNU, STZ and TMZ (Figure 1A) in various murine organs or in cell culture (Figure 1D-G). While structurally dissimilar, each of these agents is believed to generate a methyldiazonium ion as a common intermediate (Figure 1B), which reacts with DNA by an S_N_1 chemical mechanism to generate m6G. The present work defined the high-resolution mutagenic fingerprints of NDMA, TMZ, STZ and MNU across 96 3-base contexts in the mouse genome. As expected (40,41,46,47), the mutations of NDMA were primarily GC→AT transitions and were strongly enhanced in cells lacking *O*^6^-methylguanine-methyltransferase (MGMT), a direct reversal DNA repair protein that removes the methyl group from m6G, restoring undamaged guanine. The detailed mutational landscape across all 3-base contexts was the same in the presence or absence of MGMT, indicating that the repair protein did not favor some contexts over others. The data are most consistent with the conclusion that the appearance of rugged, but nearly identical, mutagenic landscapes for the four agents studied here was driven more by the sequence-selective reaction with DNA of the electrophilic methyldiazonium ion than by the sequence specificity of the repair protein MGMT. It was also found that the pattern of mutations observed for the four agents studied shows high computational similarity to the pattern of mutations seen in tissues of cancer patients treated with TMZ, a pattern known as COSMIC mutational signature SBS11 (48). Lastly, it is noteworthy that mechanistically informative data are yielded from the mouse model within ten weeks of exposure to NDMA, underscoring the value of high-resolution mutational analysis for probing the very earliest steps on the pathway to malignancy.

## MATERIALS AND METHODS

### Animals and *in vivo* procedures

Most experiments utilized C57BL/6J *gpt*Δ transgenic mice (49,50), a gift from T. Nohmi. Some experiments used *RaDR^R/R^*;*gpt^g/g^*;*Mgmt^−/−^* mice, which were created by crossing *RaDR^R/R^*;*gpt^g/g^*mice (37) with *Mgmt^−/−^* mice (51). NDMA was synthesized and characterized as previously described (52) (Fig. S1). A total dose of 10.5 mg/kg NDMA, reported to be the maximum tolerated dose (53), diluted in 0.9% saline was administered via intraperitoneal injection to neonatal mice (Figure 1D) (53). The first dose (3.5 mg/kg in 10 μl) was administered at 8 days of age, and the second dose (7 mg/kg in 20 μl) was administered at 15 days of age. Vehicle treated mice were administered equivalent volumes of saline. Mice were euthanized at ten weeks post-injection by CO_2_ inhalation. Both male and female mice were used in all experimental groups (*n* = 5) in all mouse experiments.

Mice were provided irradiated pelleted diet (Isopro RMH 3000; Purina Mills, Inc., St. Louis, MO) and filtered water *ad libitum* and were maintained in an AAALAC accredited facility. Mice were housed in static microisolator cages with autoclaved hardwood chip bedding and a nestlet. Macroenvironmental conditions included temperature maintenance at 70 ± 2°F, 30–70% humidity, and a 12:12 h light:dark cycle. Mice were free of the following murine pathogens: ecto- and endoparasites, mouse parvovirus, mouse hepatitis virus, Mouse Rotavirus, Minute Virus of Mice, Ectromelia, Sendai virus, Pneumonia virus of mice, Reovirus, Theilovirus, Lymphocytic Choriomeningitis Virus, *Mycoplasma pulmonis*, *Filobacterium rodentium*, Polyoma virus and Mouse Adenovirus. Pathogen status was verified by full serology testing using sentinel mice every six months, and with an abbreviated serology panel every two months. All animal procedures were performed with approval from the Massachusetts Institute of Technology (MIT) Committee on Animal Care (Protocols 0520-035-23 and 0821-075-24) in accordance with the *Guide for the Care and Use of Laboratory Animals 8th* Edition and AVMA Guidelines for Euthanasia of Animals: 2020 Edition.

### Cell culture

C57BL/6J *gpt*Δ mouse embryonic fibroblasts (MEFs) were established and maintained in high glucose, GlutaMAX DMEM (Gibco) supplemented with 10% FBS (VWR Life Sciences), sodium pyruvate (1 mM, Gibco), penicillin (100 IU) and streptomycin (100 μg/ml) (Corning) as previously described (40). Cells were shown to be mycoplasma-free by PCR. For growth-inhibition assays, 2.5 × 10^4^ cells were seeded in 6-well plates and incubated overnight. Cells were treated with S_N_1 alkylating agents as follows. TMZ (Sigma-Aldrich): 50, 100 and 200 μM (stock solutions in DMSO (Sigma-Aldrich)) in FBS-free complete media for 24 h. STZ (Sigma-Aldrich): 500, 1000 and 2000 μM (stock solutions in 0.1 M citrate buffer, pH 4.5) in HBSS, pH 7.4 for 6 h. MNU (Sigma-Aldrich): 125, 250 and 500 μM (stock solutions in 0.1 M citrate buffer, pH 4.5) in HBSS, pH 7.4 for 1 h. After the treatments, cells were washed in PBS (Gibco) and cultured in complete media for another 48 h. Cell growth was analyzed by determining percentages of cell numbers from treatment and untreated control groups by Coulter counter. For *gpt* assays, 4 × 10^5^ cells were seeded into 15 cm tissue culture dishes (4 dishes per group) and treated with TMZ, STZ and MNU, as described above, at 60–70% confluence. Viable cells were harvested and washed twice in PBS.

### *Gpt*Δ assay: Detection of mutational events that functionally inactivate the *gpt reporter* gene in the mouse or mouse-derived MEF genomes

The *gpt*Δ assay is the technology traditionally used with the *gpt*Δ mouse to probe for small mutations in the 459 bp *gpt* transgene, of which approximately 160 copies exist in the mouse diploid genome (50). The *gpt* gene, which is not expressed in the mammalian cells, is contained in a λ-EG10 viral cassette that can be recovered *ex vivo* through a lambda packaging reaction.

The λ-EG10 phage was packaged *in vitro* from genomic DNA using a Transpack packaging extract (Agilent Technologies) and then transfected into *E. coli* YG6020 expressing Cre-recombinase, generating a 6.4 kb plasmid carrying the *gpt* and chloramphenicol acetyltransferase genes. These bacteria were cultured on selective media containing chloramphenicol (25 μg/ml, Sigma-Aldrich) and 6-TG (25 μg/ml, Sigma-Aldrich) or chloramphenicol alone. 6-TG resistance was confirmed by regrowth of colonies on plates containing chloramphenicol and 6-TG. The mutant frequency of each group was calculated (ratio of total 6-TG-resistant colonies to the average of chloramphenicol resistant colonies). The samples were processed and analyzed in a blinded fashion.

In this study, the *gpt*Δ assay was performed on several tissues isolated from the mouse experiments. Specifically, the liver, kidneys, and lungs were collected at ten weeks following the second NDMA injection, flash frozen in liquid nitrogen, and stored at −80°C until analysis. Genomic DNA was extracted from ∼25 mg of liver tissue, one kidney, and whole lungs using the RecoverEase DNA Isolation Kit (Agilent Technologies).

The *gpt*Δ assay as described above was also performed on MEFs derived from the *gpt*Δ mouse (see above) that were treated with the toxicants studied here. For the cell culture studies, genomic DNA from 2 × 10^6^ cells per group was prepared using the MegaLong DNA Isolation Kit (G-Biosciences) according to the manufacturer's instructions.

### Duplex sequencing (DS)

To determine the identity of mutations and the sequence contexts in which they occur, we utilized a form of error-corrected sequencing called duplex sequencing (DS). Previously, we described how DS can be used directly on genomic samples derived from the *gpt*Δ mouse to yield high-resolution mutational spectra in an unbiased (unselected) manner (49). Unlike the traditional *gpt*Δ assay, DS evaluates the mutations throughout the entire 6382 bp transgenic region (which contains the *gpt* gene), without the need for a phenotypic selection. In the present work, 6.4 kb *gpt-*containing plasmids from *gpt*Δ mouse livers and cell culture were isolated using a Miniprep Kit (Qiagen) following instructions of the manufacturer. Approximately 6 μg of plasmid DNA was diluted in 1× TE buffer-low EDTA, pH 8 (Thermo Scientific), transferred to a microTUBE AFA Fiber Pre-Slit Snap-Cap (Covaris) and sonicated using a Covaris E220 Focused-ultrasonicator. The following program was used to obtain 300–350 bp fragments: peak incident power = 140 W, duty factor = 10%, cycles per burst = 200, treatment time = 80 s, temperature = 4–7°C. DS libraries for *gpt*Δ C57BL/6J mice and MEFs were prepared using the NEBNext Ultra II DNA Library Prep (E7103, NEB) or as described in Chawanthayatham et al. (49) and sequenced on a NextSeq500 or NovaSeq 6000 Illumina platform by using a 150 bp paired-end protocol (MIT BioMicro Center). DS libraries for the *gpt*Δ mouse lungs and *Mgmt*^−/−^ mice were prepared using an input of 1 μg or 500 ng genomic DNA, respectively, using a TwinStrand Biosciences, Inc. Mouse Mutagenesis Kit according to the manufacturer's protocol. This kit uses probes that hybridize to 20 genomic regions, each 2.4 kb long. The genomic coordinates are provided in Supplementary Table S1. These probes were purposely selected to map in intronic and intergenic regions, to avoid selection bias. The libraries were sequenced on an Illumina NovaSeq 6000 DNA sequencer on an S4 flow cell using a 150 bp paired-end protocol (MIT BioMicro Center).

### Data processing

For each sample, the two fastq files generated by the Illumina sequencer were converted into an unaligned bam file, using Picard Tools. The resulting bam file was processed using UnifiedConsensusMaker.py (version 3.0) as available from the Kennedy Lab at University of Washington (https://github.com/Kennedy-Lab-UW/Duplex-Sequencing). Briefly, this program queries the molecular tags on each read and assembles them into families (strands with the same tag). These are subsequently collapsed into single-strand consensus sequences (SSCS). Then, the software identifies the SSCS corresponding to the complementary strand; these are grouped together as a double-strand consensus sequence (DCS). Unpaired SSCSs were flagged and ignored for the rest of the analysis. Sequence information was accepted only when the information from the two complementary DNA strands was in perfect agreement. The DCSs (as fastq files) were subsequently aligned to the target region (6382 nt in the λ-EG10 genome) using Burrows-Wheeler aligner. Unmapped reads were filtered out. Finally, the properly aligned DCS reads were trimmed (bases 1–8 and 120–137) and collapsed into a pileup file using SamTools. The pileup file was then analyzed to identify the mutation count (CountMuts.py) and their sequence location (mut-position.py). Custom Python scripts were then used to generate a list of unique mutations (a mutation at a given genomic location is counted only once), construct, normalize and plot mutational spectra. Normalization was done by dividing the mutational frequency at each trinucleotide sequence context to the frequency of that trinucleotide sequence context in the target region. For the samples generated using the TwinStrand Biosciences Inc. kit, the analysis was performed using the manufacturer's recommended pipeline running on DNANexus. The resulting mutation-position files (.mut) were then analyzed as described above to generate the normalized mutational spectra. The relative trinucleotide frequencies in the 6.4kb target from the *gpt*Δ mouse and from the TwinStrand muta-mouse1.0 probe set are shown in Figures S2 and S3 in the *SI Appendix*.

### Histological analysis

Sections of kidney and liver from mice ten weeks post treatment were fixed in 10% buffered formalin, embedded in paraffin, and sectioned at 5 μm thickness, stained with hematoxylin and eosin (H&E) by the MIT Division of Comparative Medicine Comparative Pathology Laboratory. H&E-stained sections of liver and kidney were scored by a Board-certified veterinary pathologist blinded to sample identity. Sections of liver were evaluated for the presence of hepatocellular degeneration (cell swelling with cytoplasmic alteration), fibrosis, and lipidosis using distribution-based ordinal scoring: 0 = normal, 1, uncommon detection in < 5% of liver fields (200×); 2, detectable in up to 5–20% of liver fields; 3, detectable in up to 20–65% of liver fields; 4, detectable in >65% of liver fields. The following criteria were used for scoring hepatic inflammation, necrosis, nuclear enlargement (karyomegaly), extramedullary hematopoiesis, Kupffer cell hyperplasia, Ito cell hyperplasia, and bile duct hyperplasia: 0 = normal; 1 = minimal (1–5 foci), 2 = mild (6–12 foci), 3 = moderate (13–18 foci), 4 = severe (>18 foci) (adapted from (54)). A total inflammation score for each liver was generated by combining individual scores of portal, midzonal, and centrilobular inflammation from each submitted section. Sections of kidney were evaluated for the presence of inflammation, glomerulonephropathy, tubular necrosis, tubular degeneration, tubular regeneration, tubular casts and interstitial fibrosis. Slides were examined using an Olympus BX41 microscope attached with an iKona digital camera and photographed.

### Statistical analysis

GraphPad Prism 9 was used for statistical analyses of all data. *Gpt* mutant frequencies and histopathology scores were compared by the Mann–Whitney *U*-test. The differences between groups were considered significant when the *P* value was <0.05.

### Illustration tools

Graphical images were created with CorelDRAW 2019. The pLOGO plots were generated using Schwartz Lab probability logo generator (https://plogo.uconn.edu/).

## RESULTS

### NDMA-induced mutations and mutational patterns

**Figure 2.**
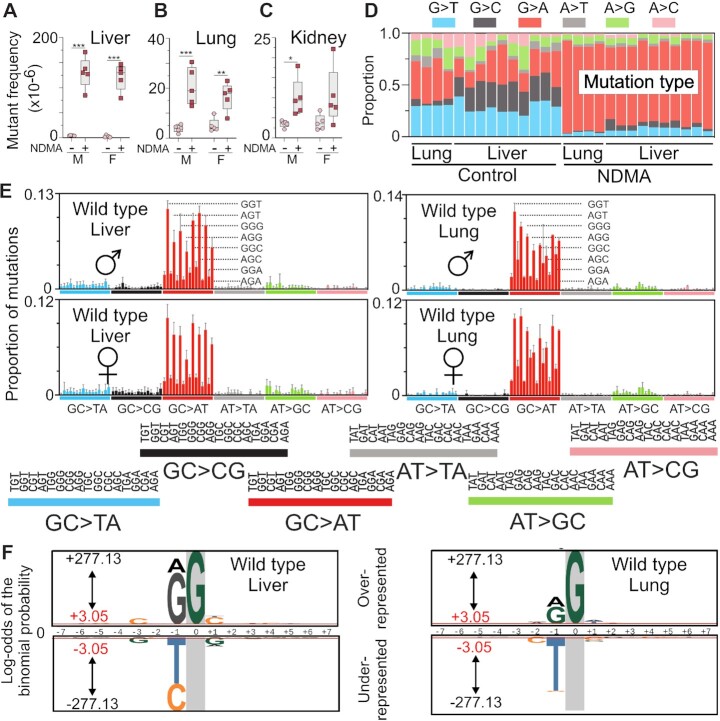
Organ-specific mutagenicity of NDMA and HRMS of NDMA-treated livers and lungs. (**A**–**C**) NDMA induced mutant frequencies of the liver, lung and kidney in *gpt*Δ C57BL/6J wild-type mice. (**D**) The proportion of single-nucleotide substitution mutations in the liver and lung was measured directly by using duplex sequencing (DS). (**E**) DS revealed the NDMA-induced high resolution mutational spectrum in liver and lung. The spectrum was dominated by GC→AT mutations with a lower frequency of AT→GC transitions. The sequencing data were plotted as the mutant base (X) accompanied by its 5’ and 3’ neighbors (N); that is, 5’-NXN-3’. There are 16 combinations for each of the six types of base substitution mutations, resulting in a total of 96 possible triplex contexts. The data shown are averaged from the livers of five males and five females and the lungs of two males and two females treated with NDMA. (F) The probability LOGO (pLOGO) was generated from all 15-base pair sequence contexts adjacent to the mutated base, GC→AT. Guanine in gray highlight represents the fixed G position. For panels A-C, statistical comparisons were done with Mann–Whitney *U*-test, *P = 0.0159, **P = 0.0079. For panel E, bars reflect averages, error bars denote 1 SD. For panel F, the 15-base sequence contexts with G→A mutations fixed at the zero position were extracted and from all datasets. Shown is the compilation of all sequence contexts. Inter-individual replicate sequences were included in the analysis. The red bar (log-odds value of ± 3.05) indicates the *P* = 0.05 statistically significant threshold following Bonferroni correction. The foreground sequences (fg) represent NDMA-induced mutations (liver *n*(fg) = 3751; lung *n*(fg) = 3451). The background sequences (bg) represent the genome sequenced (liver *n*(bg) = 13 041; lung *n*(bg) = 10 437).

To assess the mutagenic effects of NDMA *in vivo*, we used an established carcinogenic protocol (37,53) in which neonatal male and female *gpt*Δ C57BL/6J mice were administered two intraperitoneal injections of NDMA (10.5 mg/kg total, Figure 1D). Animals were aged for ten weeks following the second dose. The *gpt*Δ C57BL/6J mice carry the λ EG10 transgene with multiple copies of the *Escherichia coli gpt* gene, which was used as a reporter to detect point mutations induced by NDMA. Positive selection of mutant colonies with 6-TG allowed for quantitation of the mutant frequencies of NDMA in various tissues (Figure 1F). NDMA, at ten weeks post-exposure, resulted in increased numbers of mutations in the liver, lung and kidney (Figure 2A–C), all of which are target organs in various animal models of NDMA-induced cancer. There were significant increases of ∼30- and 45-fold in the mutant frequencies in the livers of NDMA-treated males and females, respectively, as compared to controls (Figure 2A, **P = 0.0079). Lung point mutations were also increased by >4-fold in NDMA-treated mice compared to controls (Figure 2B, males **P = 0.0079, females *P = 0.0159). Kidneys experienced an approximate doubling in the mutant frequencies in both males and females; however, only the males were found to be significantly increased (Figure 2C, **P = 0.0079).

Duplex sequencing, a very sensitive DNA sequencing method that is four orders of magnitude more accurate than conventional next-generation sequencing (49,55), was used to generate high-resolution mutational spectra from the lungs and livers of NDMA-treated *gpt*Δ mice. The base-substitution profile (Figure 2D) and high-resolution mutational spectrum (Figure 2E) of NDMA obtained by deep sequencing was dominated by GC→AT transition mutations, which were presumably caused by the mutagenic DNA adduct m6G pairing with thymine during replication *in vivo* (27,56,57). As seen in Figure 2D–E, these transition mutations were present as early as ten weeks following NDMA administration. These early-onset GC→AT mutations occurred in a distinctive pattern, with the sequence context dependency of 5’-PuGN-3’ (Fig. 2E; where Pu = a purine and N = any base). The strongly contoured mutational spectrum of NDMA was highly reproducible and did not appear to be influenced by the sex of the animal. By contrast, the HRMS of the vehicle control animals featured a diverse pattern of transition and transversion mutations with no apparent sequence context tropism, except for C→T mutations in CpG sites, which are typically associated with 5-methylcytosine deamination (*SI Appendix* Fig. S4A and C). The HRMS of the lungs displayed a similar pattern (Figure 2E and Fig. S5), despite only a 4-fold increase of NDMA induced mutations compared to controls (Figure 2B). To understand the influence of neighboring bases on NDMA induced mutagenesis, we extracted the 15-base sequence contexts with G→A mutations fixed at the zero position from all datasets (Figure 2F). The probability LOGO (pLOGO) (58) was utilized to visualize the biased appearance of adjacent bases. Guanine and adenine bases were significantly overrepresented at the 5’-proximal position to the observed G→A mutations in both the liver and lung spectra. Other significantly overrepresented bases are summarized in Tables 1 and 2. By contrast, the most frequent and significant base found at the proximal 5’-position in control (vehicle-treated) animals was cytosine (*SI Appendix* Figure S6A, B and Supplementary Table S2), which is a distinguishing molecular feature of COSMIC SBS1 (48), a common background mutational signature in which C→T mutations occur in CpG sites due to the deamination of 5-methylcytosine.

**Table 1.** The probability LOGO statistics generated from the livers of wild-type mice treated with NDMA. The value represents the log-odds binomial probability. The 15-base sequence contexts with G→A mutations fixed at the zero position were extracted from all datasets (5 males and 5 females) and compiled with inter-individual replicate sequences included in analysis

**Position**	**Frequency (%)**	**Value**
**C at -6**	28.0%	3.68
**C at -5**	26.7	4.79
**C at -3**	31.4	22.96
**G at -1**	47.4	193.23
**A at -1**	30.9	52.74
**G at 0**		
**C at 1**	38.4	27.34
**T at 1**	23.8	4.97
**A at 3**	28.4	4.00
**C at 4**	26.0	6.07

**Table 2.** The probability LOGO statistics generated from the lungs of wild-type mice treated with NDMA. The value represents the log-odds binomial probability. The 15-base sequence contexts with G→A mutations fixed at the zero position were extracted from all datasets (2 males and 2 females) and compiled with inter-individual replicate sequences included in analysis

**Position**	**Frequency (%)**	**Value**
**A at -2**	34.3	6.29
**G at -1**	39.2	64.57
**A at -1**	45.9	37.22
**G at 0**		
**A at 1**	32.2	5.89
**T at 1**	29.1	10.31
**A at 2**	32.0	4.51

### Histopathologic changes in liver consistent with xenobiotic insult

**Figure 3.**
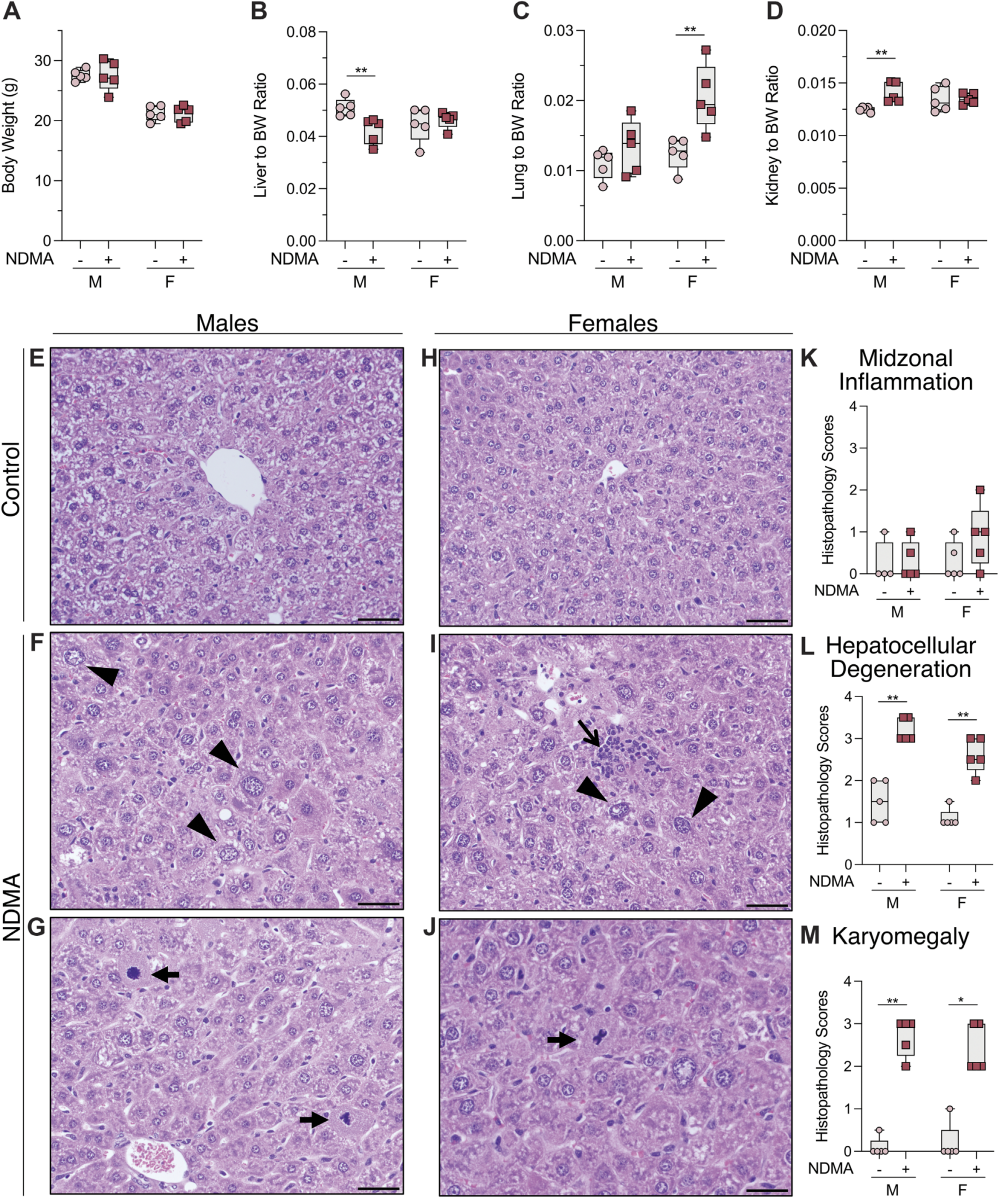
Macroscopic and histological changes at 10 weeks post NDMA treatment. (**A**) Neonatal *gptΔ* C57BL/6J mice were administered a total dose of 10.5 mg/kg NDMA split between day 8 (1/3 dose) and day 15 (2/3 dose). Samples were collected 10 weeks post-NDMA treatment. (**B**–**D**) Organ to body weight ratios. (**E**–**J**) Representative hematoxylin and eosin (H&E) sections of liver and (**K**–**M**) histopathology scores from male and female C57BL/6J *gpt*Δ mice treated with saline (control) or NDMA as described in material and methods. (F) NDMA-treated male mice exhibited areas of periacinar hepatocellular degeneration (swelling) with or without karyomegaly (arrowheads) and (G) bizarre mitotic figures (arrows). (I) Sections of the liver from an NDMA-treated female mice contained multifocal areas of hepatocellular degeneration with or without karyomegaly (arrowheads), bizarre mitoses (J, arrow), and lymphocytic and histiocytic infiltrates (open arrow). Original magnification x400, scale bars = 50 μm. Whitney *U*-test, *P = 0.0159, **P = 0.0079.

NDMA induced carcinogenesis in animal models has a higher frequency in males compared to females. However, our results demonstrate that NDMA induces an equivalent mutational burden and spectra in both sexes. Histological analysis of the liver was performed to evaluate any potential differences. At the time of euthanasia, there was no difference in body weights of treated animals compared to vehicle controls (Figure 3A). In NDMA-treated males, the liver-to-body weight ratio decreased significantly, whereas the kidney-to-body weight ratio increased (Figure 3B and D). Females treated with NDMA did not show the same trends as males, but they did show increased lung-to-body weight ratios (Figure 3C).

Macroscopic changes were not observed in any organs. Histological examination of livers from NDMA-treated mice, however, revealed multifocal areas of periacinar (centrilobular to midzonal) hepatocellular degeneration (Figure 3F–G, I–J) evidenced by hydropic degeneration (swelling) and cytoplasmic alteration (Figure 3F and 3L; **P = 0.0079) and karyomegaly (Figure 3I and 3M; **P = 0.0079). The location of these changes within the liver is consistent with the high concentration of P450s in the pericentral hepatocytes resulting in the greatest exposure to the reactive metabolite of NDMA. Occasionally, hepatocytes from NDMA-treated mice exhibited atypical mitoses (Figure 3G and J) possibly resulting in chromosome missegregation and aneuploidy associated with oncogenesis (59). Scattered within the hepatic parenchyma, there were low numbers of lymphocytic and histiocytic infiltrates in NDMA-treated mice (Figure 3I).

### Role of MGMT as a protection against NDMA *in vivo*

**Figure 4.**
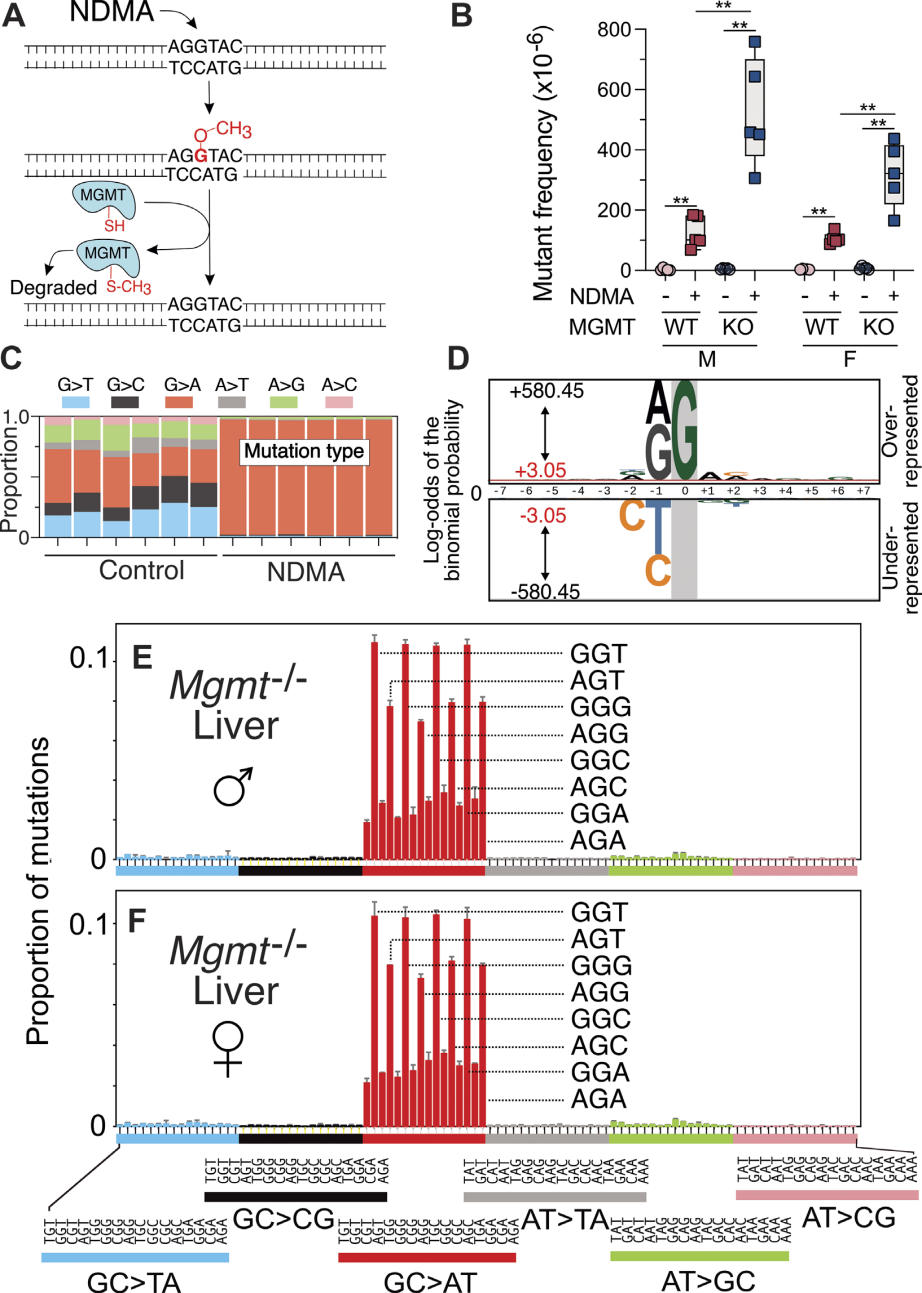
NDMA-induced mutations and mutational spectra in livers of MGMT-deficient mice. (**A**) The biochemical mechanism of MGMT, the repair protein for m6G. NDMA generates an electrophile *in vivo* that methylates DNA to form, among other DNA lesions, m6G. A nucleophilic cysteine thiol residue on MGMT attacks the methyl group of m6G, resulting in a methylated MGMT protein and an undamaged guanine. (**B**) *Mgmt^−/−^ gptΔ* C57BL/6J mice were administered NDMA (10.5 mg/kg total dose) using the regimen of Figure 1D, and liver samples were collected 10 weeks following treatment. NDMA-induced point mutant frequencies in the livers of male and female MGMT-deficient and -proficient mice are shown. (**C**) The proportion of single-nucleotide mutations in livers of saline control and NDMA-treated *Mgmt^−/−^* mice measured directly by using DS. (**D**) The pLOGO analysis produced from all 15-base pair sequence contexts adjacent to the mutated base, GC→AT. Guanine in gray highlight represents the fixed G position. (**E**, **F**) HRMS from the livers of three male and three female MGMT-deficient mice treated with NDMA. For panel B, statistical comparisons were done with the Mann–Whitney *U*-test, *P = 0.0159, **P = 0.0079. For panel D, the 15-base sequence contexts with G→A mutations fixed at the zero position were extracted from all datasets. Shown is the compilation of all sequence contexts. Inter-individual replicate sequences were included in the analysis. The red bar (log-odds value of ±3.05) indicates the *P* = 0.05 statistically significant threshold following Bonferroni correction. The foreground sequences (fg) represent NDMA-induced mutations (liver *n*(fg) = 52 253). The background sequences (bg) represent the genome sequenced (liver *n*(bg) = 10 437). For panels E and F, bars reflect averages (*n* = 3 for each group), error bars denote 1 SD.

**Figure 5.**
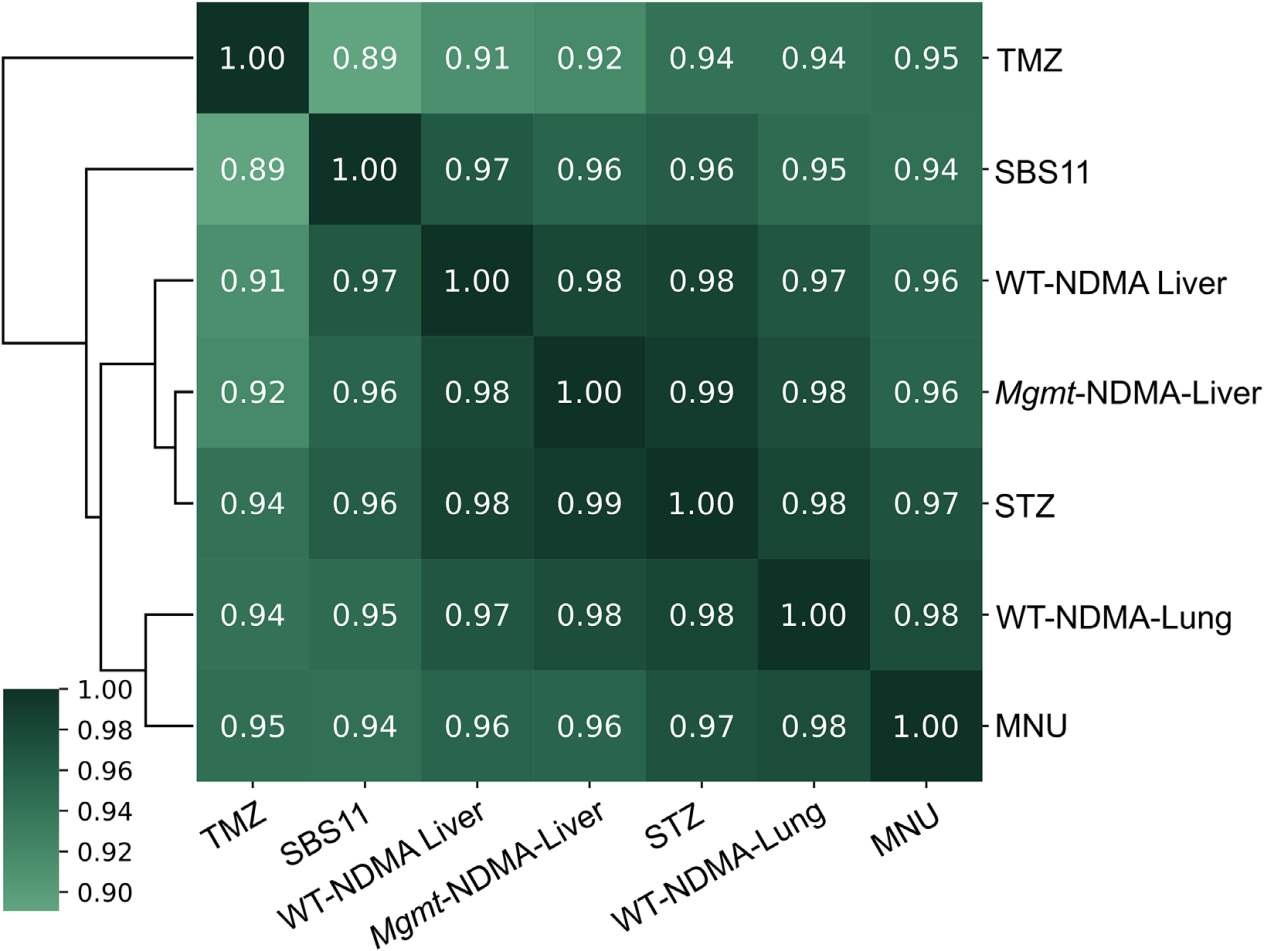
Cosine similarity matrix of the mutational spectra of NDMA treated WT liver (NDMA-WT-liver), WT lung (NDMA-WT-lung) and MGMT-deficient mice (NDMA-*Mgmt*); MEFs treated with temozolomide (TMZ), *N*-methyl-*N*-nitrosourea (MNU), and streptozotocin (STZ); and human mutational signature SBS11. Before performing the cosine similarity comparisons, all mutational spectra were baseline corrected by subtracting the corresponding background (vehicle-treated) spectrum, and then normalized to reflect mutation frequency per trinucleotide.

The DNA adduct m6G is subject to direct reversal repair by MGMT (Figure 4A), and pharmacological inhibition of this protein in cells derived from the *gpt*Δ C57BL/6J mouse results in an increase in m6G levels in DNA following methylating agent exposure (40). To assess the impact of MGMT on mutational frequency in the liver, MGMT-deficient mice (51) containing the *gpt*Δ and Rosa26 Direct Repeat (RaDR) (60) transgenes were exposed to NDMA (Figure 1D) (37). We observed a 3-fold (females) to 4-fold (males) enhanced mutant frequency in NDMA-treated *Mgmt*^−/−^ mice compared with NDMA-treated wild-type (WT) mice (Figure 4B). The mutant frequencies of NDMA in the WT *gpt*Δ and WT *gpt*Δ/RaDR mice, the two strains used in this study, were indistinguishable (*SI Appendix* Figure S7). To understand the role of DNA repair in the sequence context-dependent formation of NDMA mutations, DS was performed on genomic DNA isolated from livers of NDMA-treated *Mgmt*^−/-^ mice. As was seen in the NDMA-treated WT mice, GC→AT mutations were the dominant single-base substitution recorded at 10 weeks post-NDMA exposure, with a preferential distribution once again in the 5’-PuGN-3’ context (Figure 4D–F). Overall, the mutational patterns of NDMA in *Mgmt*^−/−^and WT mice were nearly identical, displaying a cosine similarity of 0.98 (Figure 5). In both genotypes, but most conspicuously seen in the *Mgmt*^−/-^ mice, mutations were more abundant in the 5’-GGN-3’ sequence context, as compared with the 5’-AGN-3’ context (Figure 4C, E–F; Figure 2D–E). The reason for this context specificity of mutations is unclear, but some possibilities are considered in the Discussion. The HRMS of vehicle control-treated *Mgmt*^−/−^ mice (*SI Appendix* Fig. S4B and D) showed a diverse array of background mutations like that observed in WT mice (*SI Appendix* Figure S4A and C). Taken together, the evidence points to the spectra seen in Figures 2E and 4E–F as defining features reflecting exposure to NDMA or, as shown below, to other agents that generate a methyldiazonium ion prior to reacting with DNA. Similar to the WT mice, analysis of neighboring bases revealed that guanine and adenine were significantly overrepresented at the most proximal 5’-position of the G→A mutation (Figure 4D and Table 3), whereas the control spectra frequently featured C→T mutations at the 5’ C of CpG hotpots (Figure S6C and Table S2).

**Table 3.** The probability LOGO statistics generated from the liver of *Mgmt^−/−^* mice treated with NDMA. The value represents the log-odds binomial probability. The 15-base sequence contexts with G→A mutations fixed at the zero position were extracted from all datasets (4 males and 4 females) and compiled with inter-individual replicate sequences included in analysis

**Position**	**Frequency (%)**	**Value**
**G at -6**	23.9	4.72
**G at -5**	22.8	4.67
**G at -4**	22.6	5.86
**G at -3**	22.9	4.27
**A at -3**	29.2	4.17
**A at -2**	32.8	29.44
**G at -2**	24.3	23.51
**T at -2**	24.7	9.33
**G at -1**	41.6	324
**A at -1**	42.5	256.46
**G at 0**		
**A at 1**	31.4	47.10
**A at 2**	31.0	26.02
**C at 2**	24.5	22.44
**A at 3**	29.4	12.99
**G at 4**	23.1	13.22
**A at 5**	28.3	3.94
**G at 5**	22.8	3.76
**G at 6**	24.8	20.10
**G at 7**	24.2	4.88

### Comparison of mutational patterns of NDMA with other S_N_1 alkylating agents: S_N_1 agents show cosine similarity to human mutational signature SBS11

**Figure 6.**
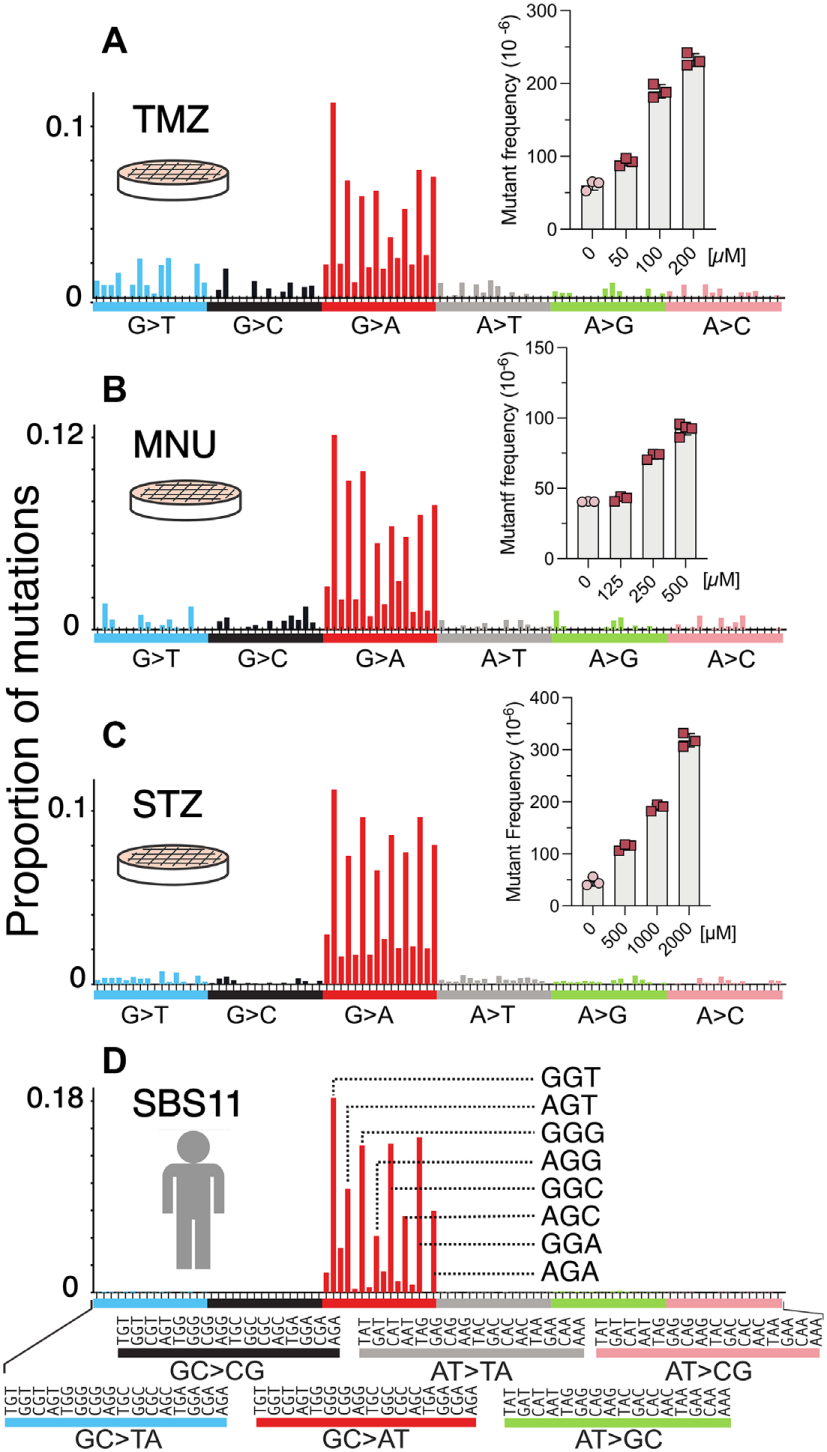
HRMS of MEFs treated with alkylating agents MNU, STZ and TMZ. The plots depict the HRMS data and their dose-dependent mutagenicities at the *gpt* locus (insets) for MNU (**A**), STZ (**B**) and TMZ (**C**). (**D**) COSMIC mutational signature SBS11 of human cancer.

Animal models are an indispensable tool for recapitulating the toxicological effects of environmental agents in humans. However, to reduce the number of animals utilized in research and make our research more efficient, we have generated a mouse embryonic fibroblast cell line from the *gpt*Δ mice (40). With the understanding that cell culture models lack multiple *in vivo* features, such as xenobiotic metabolism, and tissue distribution and exposure, we set out to analyze several S_N_1 alkylating agents of clinical relevance that do not require metabolic activation (MNU, STZ, TMZ, all of which are or have been used in cancer therapy). To investigate these structurally diverse, but similar-acting alkylating agents, we first performed a dose-response toxicity study in the MEFs. Cells treated with doses generating no more than 50% growth inhibition were subsequently collected for mutational analyses, and HRMS were generated for each of the compounds (Figure 6A–C). The mutational spectra of all three alkylating agents predominantly displayed GC→AT mutations, with a pattern strongly resembling the mutational spectrum of NDMA (Figures 2E and 4E, F). The mutations of MNU, STZ and TMZ were primarily in the 5’-PuGN-3’ sequence context (Figure 6A–C); their detailed spectra displayed a high cosine similarity (>0.9) with each other, with the NDMA spectra from WT animals (in both liver and lung), with the spectra from the livers of MGMT-deficient animals, as well as with COSMIC mutational signature SBS11 (Figure 5). COSMIC mutational signature SBS11 (Figure 6D) is a mathematically extracted pattern from the sequences of thousands of human tumors, and is found in samples from patients that were treated with S_N_1 therapeutic agents, such as temozolomide and dacarbazine (48). These spectra are also markedly different (cosine similarity < 0.5) than the background mutational spectra in each of the experimental systems described here (i.e. mouse liver, lung, MEFs) (Figure S8).

## DISCUSSION

There is a great need for biomarkers that appear in the initial stages of the carcinogenic process. From a practical perspective, early onset biomarkers could invoke increased monitoring or intervention by surgery, or other forms of treatment that serve to thwart tumor development. From a mechanistic perspective, such biomarkers provide insight into the biochemical and genetic events that lead to disease. Understanding those events is an important step towards establishing mechanistically informed novel cancer prevention and treatment strategies. The present work is based on a classical view of chemical carcinogenesis as involving an early phase, during which DNA adducts of the carcinogen trigger a founder or initiation spectrum of mutations, followed by a long period, during which further genetic and non-genetic events lead to cancer development. During tumor outgrowth, promotional events, such as an activated immune system, cause additional diversity among the heterogeneous population of cells in the malignant mass. As shown here with NDMA, and in work on other agents (49,61,62), the founder spectrum can be a highly distinctive genetic fingerprint. Indeed, that fingerprint could help to identify which agents, among the many to which people are exposed, contribute to the causation of specific human cancers. Of note is the fact that with NDMA, and in earlier work on the liver carcinogen, aflatoxin B_1_ (49), the founder spectrum appears as early as ten weeks into the carcinogenic process—well before morphological abnormalities develop—offering the possibility that the founder spectrum can be used as a harbinger of subsequent disease. In an Adverse Outcome Pathway (AOP) framework, the early appearance of the founder spectrum constitutes a key event that correlates with the increased risk of the adverse outcome, which is cancer. Liquid biopsies, where circulating mutant DNA or cells are analyzed, offer one avenue by which this predictive goal could be realized.

Examination of the high-resolution mutational spectrum of NDMA reveals a rugged, distinctive mutational landscape (Figures 2 and 4). For NDMA, as well as for other mutagenic carcinogens, the distribution of point mutations across sequence space reflects the chemical steps that generate the observed mutational spectrum. We have proposed that, in general, three chemical factors mold the sequence-dependent mutational patterns: 1) DNA lesion formation, 2) lesion repair, and 3) error-prone replication across the DNA lesion (63). When considering these mechanistic elements, it is not surprising that the mutational spectra of NDMA and the three clinical agents (MNU, STZ and TMZ) reported in this study were, in fact, highly similar. Each is a methylating agent capable of forming the same reactive electrophile that reacts with DNA by an S_N_1 chemical mechanism. In sum, the mutational spectra we observed reflect the properties of the methyldiazonium ion in cells. It is notable that a reaction of DNA with this ion can generate many different DNA lesions (detailed in the Introduction), but the genetic evidence seen in the present work is that the chemical modification responsible for the bulk of mutagenesis is m6G. As discussed in more detail below, this notion is reinforced by the observation that the mutational frequency in liver increases strikingly in *Mgmt*^−/−^ animals treated with NDMA, and the primary client adduct for this repair protein is m6G. The impact of MGMT on mutational frequency, the dominance of GC→AT mutations, and the scarcity of all other types of point mutations in the HRMS indicate that the mutagenic contributions of other DNA lesions known to be present (e.g. m3A, *O*^4^-methylthymine (m4T), and the abasic site formed by m7G depurination) are very minor under the conditions of this experiment.

As indicated above, comparison of mutational spectra also provides mechanistic insight into the contribution of the repair protein MGMT to the NDMA-induced mutational landscape. Since the distribution of the mutations in the GC→AT region across all 16 m6G 3-base contexts is largely unchanged in *Mgmt*^−/−^ and WT mice (Figure 5, cosine similarity 0.98), it is inferred that the biochemical activity of MGMT does not have a strong sequence context specificity. Moreover, the qualitative agreement of the mutational spectra for S_N_1 methylating compounds between our mouse model and *in vitro* systems indicates that the MEF cell model provides an accurate representation of mutagenic processes *in vivo*. It is noteworthy that the mutational spectra we recorded for MNU and TMZ in MEFs are markedly different than the spectra reported for the same compounds (at similar doses) in several other studies (64,65). The mutational spectra of MNU and TMZ in human induced pluripotent stem cells were dominated by AT→GC mutations, while featuring very few GC→AT mutations (64). This difference may reflect high levels of MGMT in those cell lines. A second cell-based study of TMZ-induced mutations found an SBS11-like mutational spectrum only in tumor-derived cells that were deficient in mismatch repair (MMR) (65), suggesting that MMR deficiency may be a mechanistic requirement for SBS11. While MMR deficiency is an established survival mechanism by which cancer cells evolve resistance to alkylating agents such as TMZ (65), it is unlikely to be the only pathway that allows cells to survive alkylation damage (66,67). In the present work, while the mouse livers or MEFs were exposed to substantial doses of alkylating agents, cell toxicity was not high enough to select for MMR deficiency. Additionally, our DS methodology is applied directly to a heterogenous population of cells, without any clonal amplification, which once again prevents the selection of an unusually resistant, MMR-deficient clone. Moreover, the mutational spectra we recorded do not show discernible contributions from MMR-associated COSMIC mutational signatures (i.e. SBS6, 14, 15, 20, 21, 26 and 44), supporting the view that the SBS11-like mutational spectra we observed were unlikely to reflect MMR deficiency in addition to alkylating agent exposure.

Because earlier work in *E. coli* indicates that mutant frequency from polymerase-mediated replication past m6G is essentially identical in all 16 three-base contexts (57), and we see here that expression of MGMT affects the amount, but not the distribution of mutations, we conclude that the molecular origin of the patterns of mutations observed in the NDMA spectrum is primarily the sequence context-dependent formation of m6G. This formation follows the preferential binding and reactivity of the putative methyldiazonium ion with guanines in different DNA sequence contexts. An examination of the literature on the reaction of electrophilic methylating agents with naked DNA (68–70) is roughly in accord with the current observations *in vivo*. Given this rationale, even among the mutational hotspots at 5’-PuGN-3’ sequences, the observed increased probability of mutations at the 5’-GGN-3’ sites relative to the 5’-AGN-3’ sites could be attributed to differential chemical reactivity. The molecular basis for this effect, however, remains to be elucidated and warrants further investigation.

While the mutational spectrum of NDMA is dominated by the GC→AT mutations, it does feature a small fraction of AT→GC mutations in a pattern that cannot be explained by the background spectra (mutational spectra in vehicle-treated mice). After background subtraction, AT→GC mutations constitute 4.6% of the total number of mutations. Among the adenine and thymine lesions formed by S_N_1 alkylating agents, m3A and m4T are chemically the most likely to contribute to the observed, but infrequent, AT→GC mutations. While m3A is primarily known as a strong replication blocker, it can also be mutagenic; recent work from this laboratory shows that mice knocked out for AAG (a.k.a MPG), a glycosylase known to have m3A as its principal target, display an enhanced mutant frequency over wild-type mice when exposed to NDMA (37). Future examination of the mutational spectra of AAG-deficient mice may reveal an increase in the fraction of mutations at A:T base pairs, which would support the possible role of AAG as a protection against those mutations. A second plausible candidate for the lesion responsible for AT→GC mutations is m4T. Compared to other methylated bases formed by S_N_1 agents, the amount of m4T formed by S_N_1 alkylating agents is small (<1%). However, *in vitro* and *in vivo* studies have shown that m4T primarily leads to AT→GC mutations (71,72), suggesting that this adduct, qualitatively, has the mutagenic specificity to explain the AT→GC pattern in the NDMA-treated mice. Interestingly, as compared to WT mice, the mutational spectrum of NDMA in *Mgmt*^−/-^ mice has a significantly reduced proportion of AT→GC mutations (1.7%). This lower fraction, however, is primarily a reflection of the increased proportion of GC→AT mutations attributable to an enhanced yield of m6G in the MGMT-deleted mice.

One variable investigated in this work was the role of sex in the susceptibility of mice to the early-stage mutagenic properties of NDMA. As with most animal models, as well as humans, males are more susceptible than females to the eventual development of cancer (53,73–75). It is noteworthy that the ten-week post-dosing mutation frequencies in the livers of male and female mice treated with NDMA were similar (Figure 2), which is in contrast to the high incidence of hepatic tumors in males and their lower frequency in females reported at much later time points in the literature (53,73,75). Using a similar model system and dosing regimen, we observed the lack of a sex-based difference in mutational load with the environmental hepatocarcinogen aflatoxin B_1_, which also shows a male-selective carcinogenic tropism (76).

The reasons for the greater carcinogenic potency of NDMA and aflatoxin to males as compared to females, despite identical mutation frequencies at ten weeks post initiation, is unknown, but hints are offered from work on a structurally similar nitrosamine, diethylnitrosamine (77). In that case, the sex-based disparity has been attributed to increased levels of the pleiotropic cytokine interleukin-6 (IL-6) in males, and an estrogen-mediated inhibition of IL-6 in females (78–80). With regard to the present work, it is possible that a differential inflammatory response in adult males versus females explains the sex-based differential in cancer generation in NDMA-treated animals. Cognizant of the possible role of inflammation in tumorigenesis, we looked for, but found no histologic evidence of a greater inflammatory response to NDMA in males as compared to females at ten weeks following treatment (Figure 3). This result points to the importance of post-initiation intrinsic and extrinsic factors that are probably necessary for tumor development once the stage is set by the initial mutations produced by NDMA exposure at a young age. During a hypothetical inflammatory stage, DNA-damaging products such as reactive oxygen species (ROS) and other endogenous mutagens may be generated, and enhanced production of these species could superimpose a layer of inflammatory genomic damage on top of the damage previously acquired from toxicant-DNA interactions. As one example, ROS produce 7,8-dihydro-8-oxoguanine lesions, which, when left unrepaired, result in GC→TA mutations that accumulate in a pattern known as mutational signature SBS18 (48). Analysis of the NDMA mutational patterns at ten weeks did not show evidence of SBS18 or other mutational patterns typically associated with inflammation (48,64), suggesting that additional mutational processes are needed for full malignant transformation to occur following puberty (81). It is reasoned that the combination of NDMA alkylation and subsequent inflammatory DNA lesions, or the induction of inflammation-associated deaminases (82), results in the high susceptibility of male mice during carcinogenesis. Thus, while we have identified a pattern of mutations consistent with the miscoding properties of m6G at ten weeks after exposure, we would expect to see an increasing complexity in the mutational pattern during tumor development (83). That complexity was indeed was seen in our earlier work on aflatoxin B_1_ carcinogenesis (49).

While our data do not explain the well-known sex-based difference in susceptibility of males to cancer, they do help to explain the organotropism of NDMA, which induces cancer in several different organ systems. We employed a neonatal mouse model (53), in which NDMA administration to C57BL/6J male mice results in nearly 80% of the mice developing hepatic tumors, along with a 7% incidence of lung tumors. This organotropism for NDMA-induced tumors is consistent in the *gpt* mutant frequency observed in our transgenic *gpt*Δ C57BL/6J mice (Figure 2A–C), with a much higher frequency of mutations in the liver as compared to the lungs and kidneys (44,45). These differences in organ susceptibility probably reflect the differences in CYP2E1 expression, with expression significantly higher in the liver compared to kidneys and lung (84,85), and the details of the methods used for NDMA administration, including timing, dose, and route. Regarding CYP2E1 expression, it is noteworthy that this enzyme is significantly over-expressed in Type 2 diabetics (86), potentially putting them at increased risk to the genetic effects of NDMA. To compound the problem, NDMA has been reported to contaminate some batches of metformin (13), a leading drug used to control Type 2 and gestational diabetes.

## CONCLUSION

The present work on NDMA was inspired by the potential role that this toxicant plays in environmental carcinogenesis. We observed, however, that the mutational spectrum of NDMA in mice shows high similarity to human mutational signature SBS11, which has been linked to the aftereffects of cancer chemotherapy in humans (48) and to exposure of animals to certain other alkylating agents (87,88). Using MEFs derived from the same mouse used for the NDMA study, we confirmed that several agents that are used or have been used in chemotherapy, MNU, STZ and TMZ, produce the same spectrum as NDMA. When viewed as a whole, our work and that of others supports the model that SBS11 reflects a mutational process driven by S_N_1 alkylating agents that produce m6G. Hence, the observation of an SBS11-like spectrum could reflect exposure to a host of different methylating agents, including those present as environmental contaminants as well as those used in therapy. An additional observation is that the distinctive mutational spectrum of NDMA is evident very early, only ten weeks after carcinogen administration. Accordingly, it certainly reflects past exposure to DNA alkylating agents and, if it is a durable pattern and appears at the time of tumor development, it could be useful as a biomarker of later-life disease. Further, the work underscores the quantitative importance of the repair protein MGMT as a protection against these agents. And, lastly, the data are most consistent with a model whereby the distinctive mutational landscape of these agents is dictated by the preference for reaction of the DNA-reactive putative methyldiazonium ion with specific sequence contexts, most notably 5’-PuG-3’.

## DATA AVAILABILITY

The data underlying this article are available in the National Center for Biotechnology Information (NCBI) Sequence Read Archive (SRA) repository, under the BioProject PRJNA910200 (http://www.ncbi.nlm.nih.gov/bioproject/910200), and on FAIRDOMHub (https://fairdomhub.org/studies/1130). The code used for data analysis is available at the Zenodo DOI: 10.5281/zenodo.7711071.

## Supplementary Material

zcad015_Supplemental_File
