# Supplementary material for: Molecular origins of mutational spectra produced by the environmental carcinogen N-nitrosodimethylamine and SN1 chemotherapeutic agents
Source: NAR Cancer. 2023 Mar 27;5(2):zcad015. doi: 10.1093/narcan/zcad015 (PMC10041537; doi:10.1093/narcan/zcad015)
Supplement: zcad015_Supplemental_File [file zcad015_supplemental_file.docx]

**Supporting Information for**

Molecular origins of mutational spectra produced by the environmental carcinogen *N*-nitrosodimethylamine and S_N_1 chemotherapeutic agents

Amanda L. Armijo^a,b,c,e,1^, Pennapa Thongararm^a,b,c,1^, Bogdan I. Fedeles^a,b,c,1^, Judy Yau^a,b,c^, Jennifer E. Kay^b,c^, Joshua J. Corrigan^b,c^, Marisa Chancharoen^a,b,c^, Supawadee Chawanthayatham^a,b,c^, Leona D. Samson^b,c,d^, Sebastian E. Carrasco^e,f,g,h^, Bevin P. Engelward^b,c^, James G. Fox^b,c,e^, Robert G. Croy^a,b,c^ & John M. Essigmann^a,b,c^

Corresponding author: John M. Essigmann

**Email:**  [jessig@mit.edu](mailto:jessig@mit.edu)

**This PDF file includes:**

Figures S1 to S8

Tables S1 to S2

**Supplemental Figures**

**
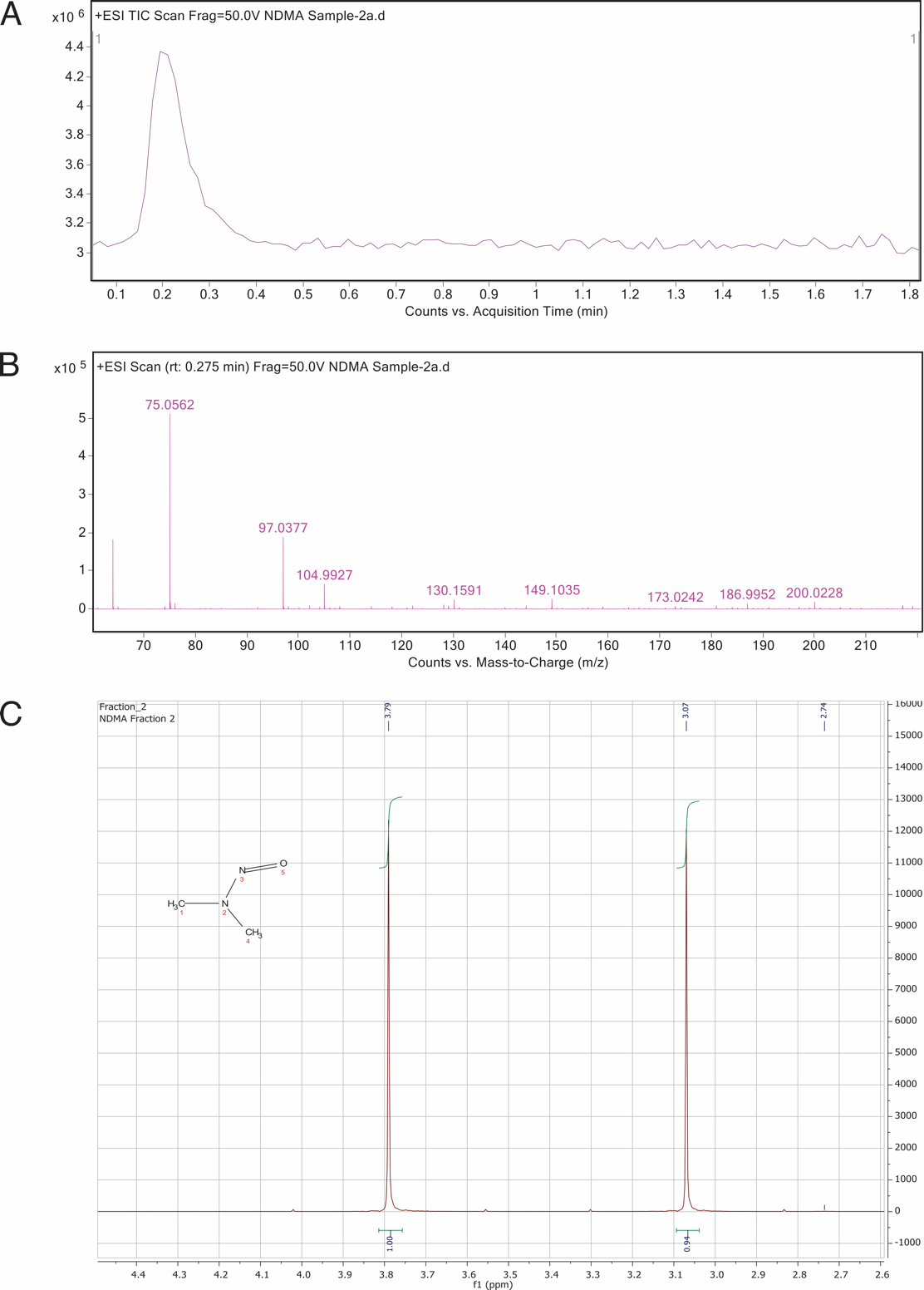
**

**Figure S1.** To establish purity of the NDMA utilized in these studies, mass spectrometry and NMR analysis were performed. (A) Total ion chromatogram and (B) mass spectrum of NDMA. The parent ion at m/z=75.0562 corresponds to the protonated NDMA ion ([NDMA-H]^+^). (C) ^1^H-NMR spectra of NDMA at 300mHz. The purity of the synthesized NDMA was >99%.


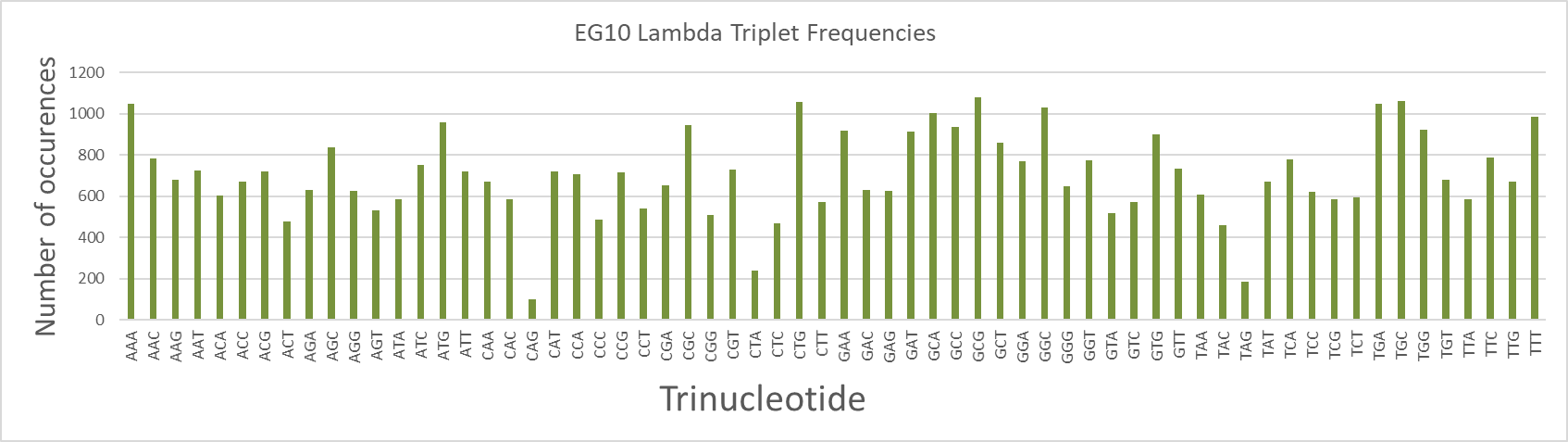


Figure S2. The number of occurrences of each trinucleotide sequence context in the ~45kb of the EG10 lambda transgenic region present in the *gpt*Δ mice. These numbers were used to normalize per-trinucleotide the mutational spectra obtained by duplex sequencing of the gpt-EG10 transgenic region.


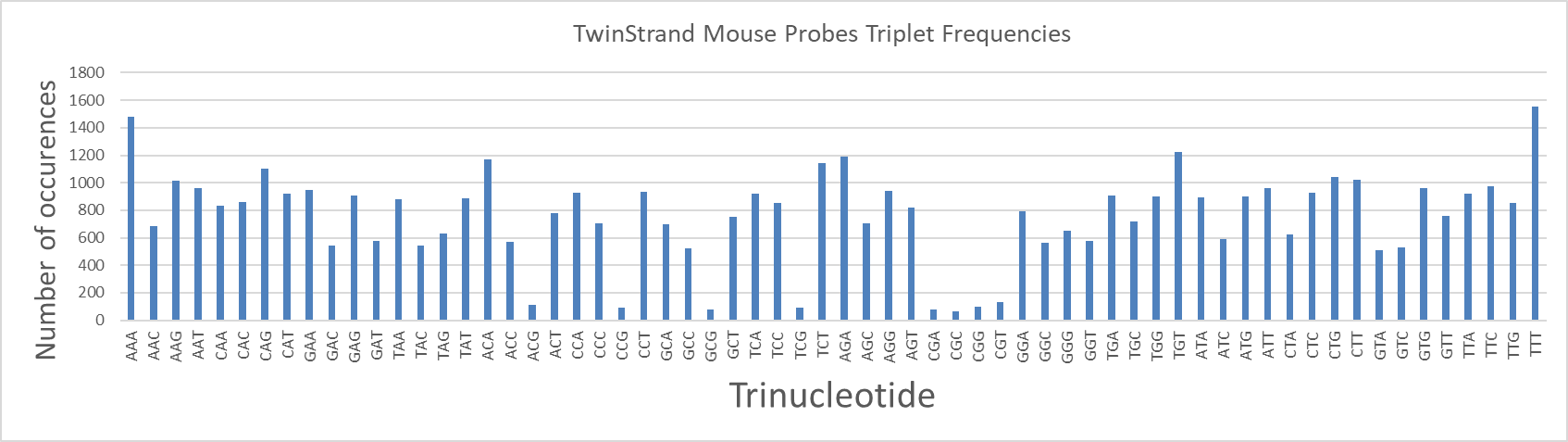


Figure S3. The number of occurrences of each trinucleotide sequence context in the 48kb of the mouse genomic regions hybrid captured and sequenced by the TwinStrand Duplex Sequencing assay kit. These numbers were used to normalize per-trinucleotide the mutational spectra obtained by duplex sequencing with the Twin Strand kit.


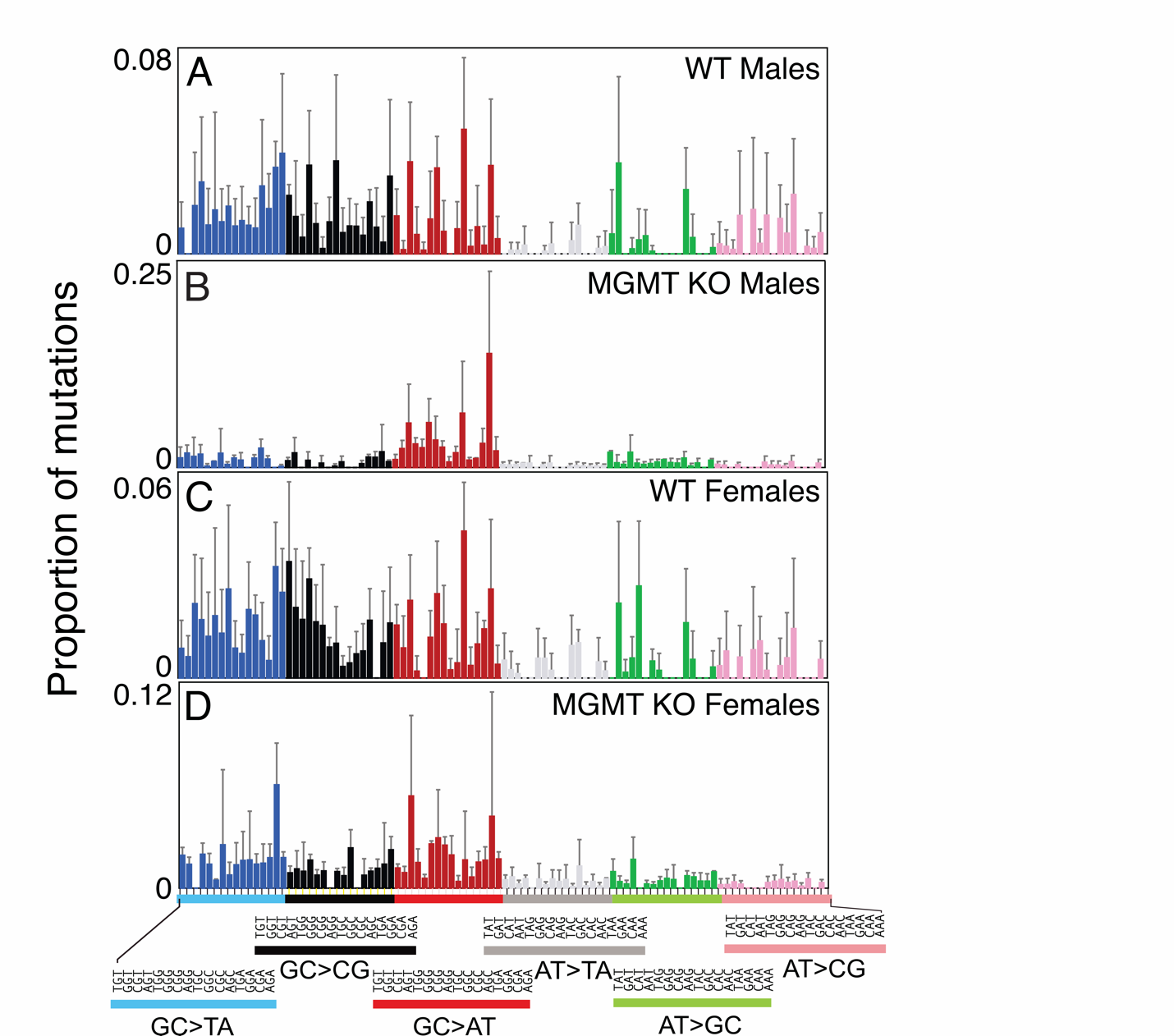


**Figure S4.** Comparison of HRMS from the liver of saline controls in (A) WT males, (B) MGMT deficient males, (C) WT females, and (D) MGMT deficient females. Bars indicate averages of n=5 (for WT mice) and n=3 for (MGMT KO mice), with error bars indicating one SD.

**Figure S5.** HRMS from the lung of saline controls in WT males and females. Bars indicate averages of n=4 with error bars indicating one SD.


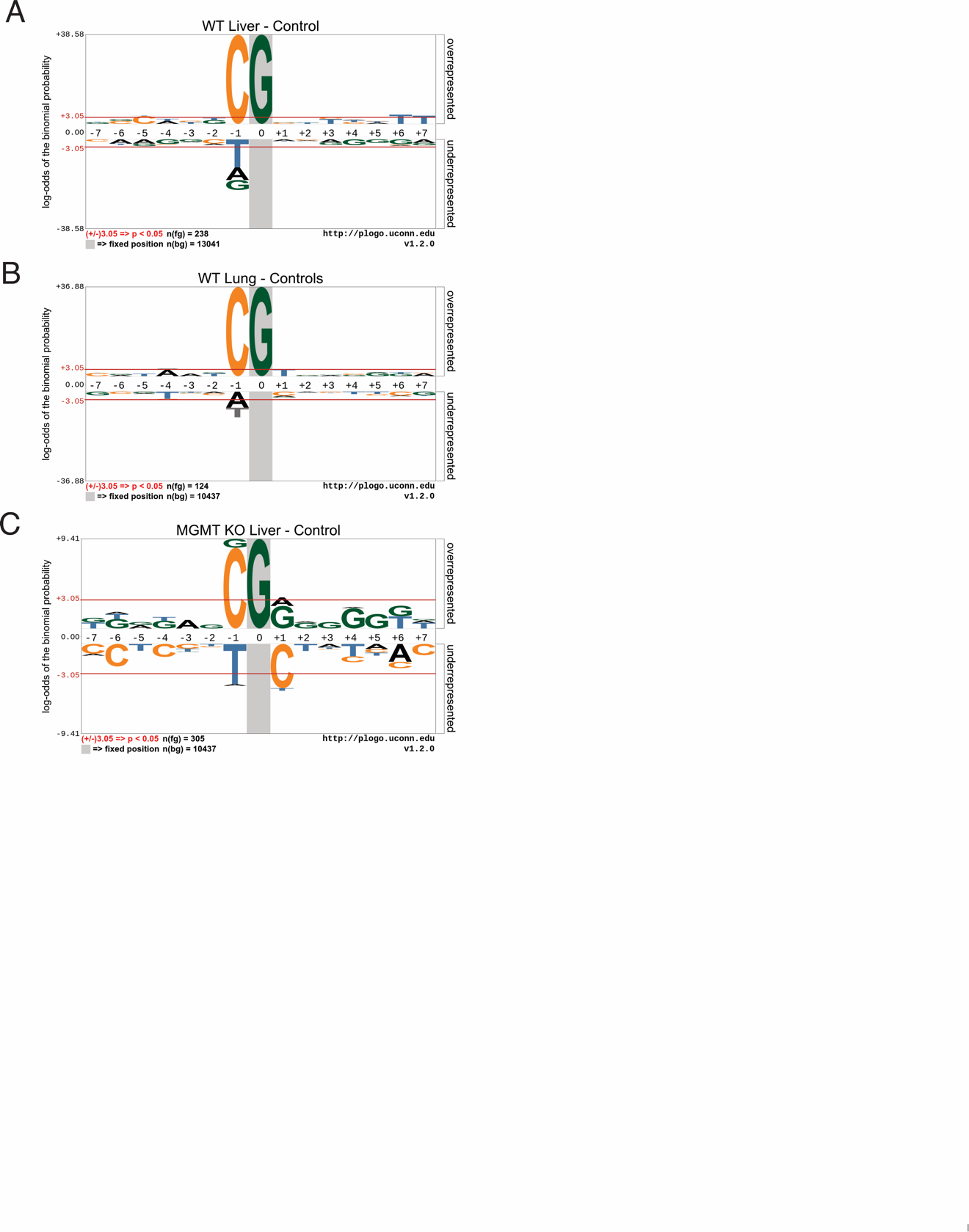


**Figure S6.** The probability LOGO of control treated animals. The probability LOGO produced from all 15-base pair sequence contexts adjacent to the mutated base, GC🡪AT. (A) Liver from WT animals (5 males and 5 females), (B) lung from WT animals (5 males and 5 females), and (C) liver from MGMT deficient animals (4 males and 4 females). The 15-base sequence contexts with G🡪A mutations fixed at the zero position were extracted from all datasets. Shown is the compilation of all sequence contexts with inter-individual replicate sequences included in analysis.


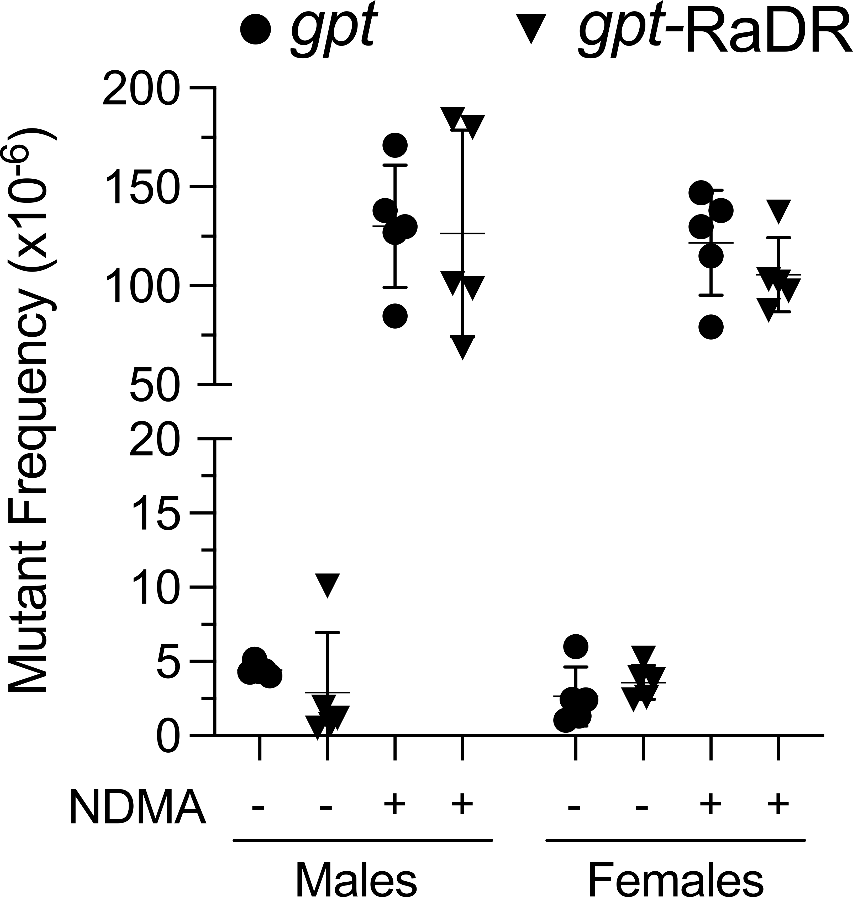


**Figure S7.** Comparison of mutational frequency between *gpt*Δ C57BL/6J and *gpt*Δ-RaDR C57BL/6J mice, as assayed with the traditional *gpt* assay. The mice were treated with either NDMA (+) or saline (vehicle control) (-). No statistical difference was observed between the two mouse strains in any of the treatment groups.


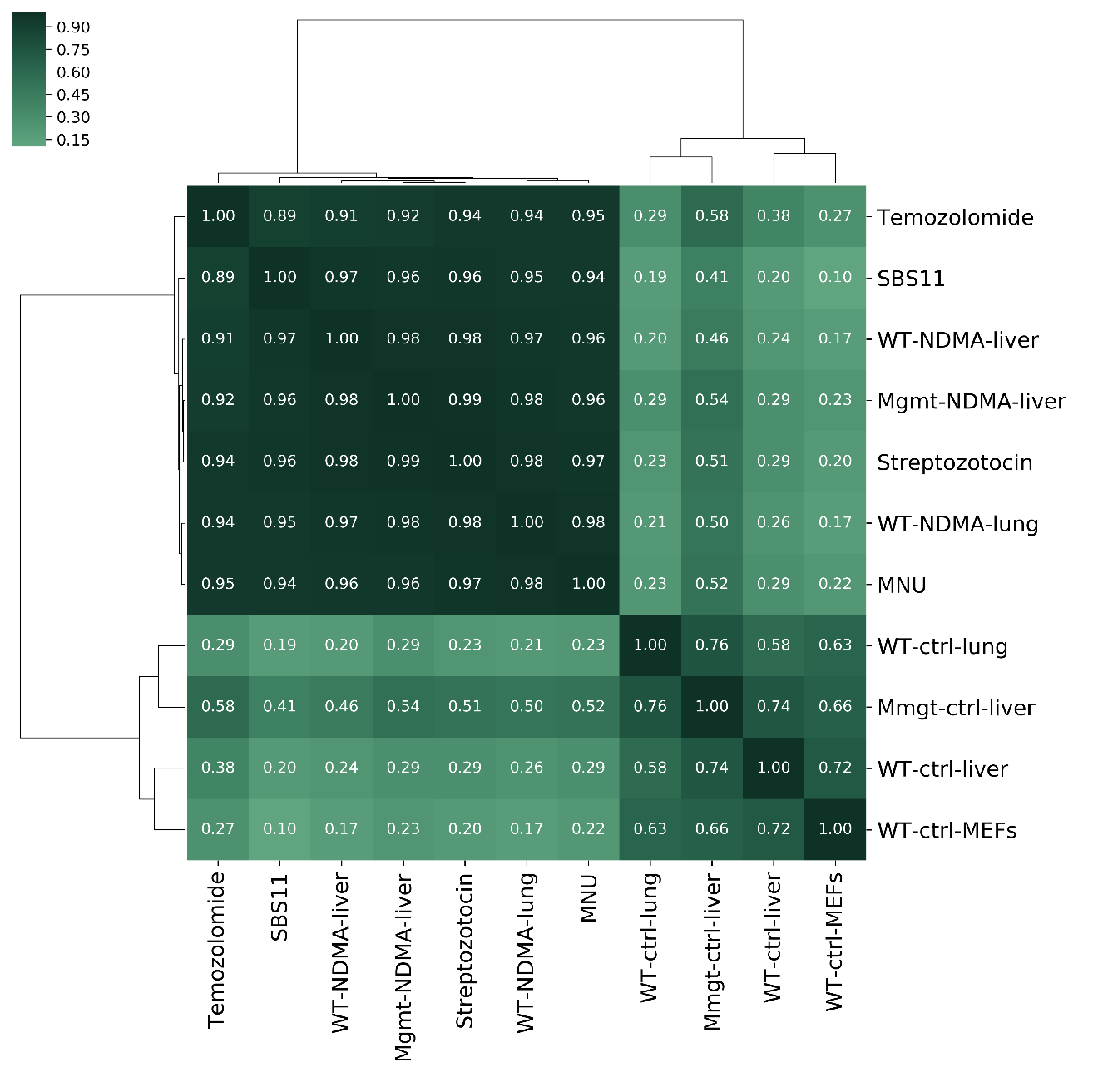


**Figure S8.** Unsupervised clustering and cosine similarity of mutational spectra generated in the present study. All alkylating agents generate highly similar spectra that cluster together (cosine similarity >0.9). The control spectra cluster together more loosely, whereas the cosine similarity between treated and untreated spectra is generally very low (<0.5), indicating no similarity.

**Table S1. The genomic coordinates of the hybrid-captured regions in the TwinStrand protocol using the panel mouse-muta-v1.0.**

| Chromosome | Start nucleotide | End nucleotide | Type of region | Gene |
| --- | --- | --- | --- | --- |
| **1** | 69304217 | 69306617 | intergenic | N/A |
| **1** | 155235938 | 155238338 | genic | Xpr1, intron 2 |
| **2** | 50833175 | 50835575 | intergenic | N/A |
| **3** | 109633160 | 109635560 | intergenic | N/A |
| **4** | 96825280 | 96827680 | intergenic | N/A |
| **5** | 18210612 | 18213012 | genic | Gnat3, intron 5 |
| **6** | 119170706 | 119173106 | genic | Cacna1c, intron 1 |
| **7** | 142683053 | 142685453 | genic | Kcnq1, intron 1 |
| **8** | 43954521 | 43956921 | intergenic | N/A |
| **9** | 28648072 | 28650472 | genic | Opcml, intron 3 |
| 10 | 21442014 | 21444414 | intergenic | N/A |
| 11 | 37934364 | 37936764 | intergenic | N/A |
| 12 | 80601002 | 80603942 | genic | Galnt16, intron 2 |
| 13 | 74030071 | 74032471 | intergenic | N/A |
| 14 | 13076171 | 13078571 | intergenic | N/A |
| 15 | 66779762 | 66782162 | genic | Ccn4, intron 2 |
| 16 | 72381580 | 72383980 | intergenic | N/A |
| 17 | 94009028 | 94011428 | intergenic | N/A |
| 18 | 81262078 | 81264478 | intergenic | N/A |
| 19 | 4618813 | 4621213 | genic | Pcx, intron 2 |

| Position | Genotype | Tissue | Frequency (%) | Value |
| --- | --- | --- | --- | --- |
| C at -5 | WT | Liver | 33.6 | 3.40 |
| C at -1 | WT | Liver | 66.8 | 35.58 |
|  | WT | Lung | 40.3 | 36.88 |
|  | *Mgmt^-/-^* | Liver | 11.8 | 8.48 |
| G at 0 |  |  |  |  |
| T at 6 | WT | Liver | 33.1 | 4.08 |
| T at 7 | WT | Liver | 34.4 | 3.61 |

**Table S2.** The probability LOGO statistics generated from control treated animals. The value represents the log-odds binomial probability. Control (saline) treated animals WT animals (liver: 5 males and 5 females, lung: 2 males and 2 females) and MGMT deficient mice (liver: 4 males and 4 females). The 15-base sequence contexts with G🡪A mutations fixed at the zero position were extracted from all datasets and compiled with inter-individual replicate sequences included in analysis.
